# Favipiravir Effectiveness and Safety in Hospitalized Moderate-Severe COVID-19 Patients: Observational Prospective Multicenter Investigation in Saudi Arabia

**DOI:** 10.3389/fmed.2022.826247

**Published:** 2022-03-04

**Authors:** Saleh Al-Muhsen, Nouf S. Al-Numair, Narjes Saheb Sharif-Askari, Roaa Basamh, Banan Alyounes, Amjad Jabaan, Fatemeh Saheb Sharif-Askari, Mohammed F. Alosaimi, Fahad Alsohime, Rabih Halwani, Haya Al-Saud

**Affiliations:** ^1^Immunology Research Laboratory, Department of Pediatrics, College of Medicine and King Saud University Medical City, King Saud University, Riyadh, Saudi Arabia; ^2^The Saudi Ministry of Health and Center of Genomic Medicine, King Faisal Specialist Hospital and Research Center, Riyadh, Saudi Arabia; ^3^Sharjah Institute of Medical Research, University of Sharjah, Sharjah, United Arab Emirates; ^4^King Abdulaziz City for Science and Technology, Riyadh, Saudi Arabia; ^5^Department of Clinical Sciences, College of Medicine, University of Sharjah, Sharjah, United Arab Emirates; ^6^Hevolution Foundation, Riyadh, Saudi Arabia

**Keywords:** COVID-19, SARS-CoV-2, favipiravir, in-hospital mortality, length of hospital stay

## Abstract

**Objectives:**

There are limited data on the efficacy and safety of favipiravir antiviral in coronavirus disease 2019 (COVID-19), particularly in the more progressed disease phase. This study aims to evaluate the favipiravir effect on reducing the length of hospital stay and in-hospital mortality among moderate and severe hospitalized COVID-19 patients.

**Methods:**

A prospective, multicenter observational study was conducted that included moderate and severe hospitalized adult COVID-19 patients in four major regions (Riyadh (Riyadh), Eastern (Dammam), Al-Qassem (Buraydah), and Macca (Jeddah) of Saudi Arabia. For the primary outcome of all-cause mortality, a Cox proportional hazard analysis was performed. While the association between favipiravir use and length of hospital stay was determined using adjusted generalized linear model. This study was approved by the Central Institutional Review Board in The Saudi Ministry of Health (MoH) with the approval number IRB # 20-85-M.

**Results:**

This study included 598 moderate and severe COVID-19 patients, of whom 156 (26%) received favipiravir. Favipiravir treatment was associated with more extended hospital stays (14 vs. 10 median days, *P* = 0.034) and higher mortality rate (aHR 3.63; 95% CI 1.06–12.45) compared to no favipiravir regimen. Despite lack of effectiveness, favipiravir use was only associated with higher diarrhea adverse effects (12 vs. 5%, *P* = 0.002), but it did not affect the renal and liver profiles of patients.

**Conclusion:**

Favipiravir was ineffective in reducing the length of hospital stay and in-hospital mortality in patients with moderate and severe COVID-19.

## Introduction

Severe acute respiratory syndrome coronavirus 2 (SARS-CoV-2) is a pathogenic coronavirus that emerged in late 2019 and has caused a pandemic of coronavirus disease 2019 (COVID-19), which has urged for development of novel therapeutics and repurposing of existing drugs. Since the start of the pandemic, national and global protocols for COVID-19 treatment have kept evolving with the continuous flow of new scientific evidence and research data.

Favipiravir is an example of an oral antiviral drug repurposed for COVID-19 management (1). It inhibits the RNA-dependent RNA polymerase (RdRp) of RNA viruses, preventing viral genome replication. In 2014, Japan approved favipiravir to treat influenza strains resistant to current antiviral medication (2). Besides COVID-19, this investigational antiviral has also been considered to manage other viral outbreaks such as Ebola (3) and the Lassa virus (4).

There are limited data on the efficacy and safety of favipiravir use in COVID-19. It has been given restricted authorization in several countries, including India and Russia, while it is under investigation in the USA, Japan and elsewhere (5). Although favipiravir is an oral medication that has shown positive clinical effects in milder cases of COVID-19 disease (6), its clinical effect in more advanced moderate and severe COVID-19 has been inconsistent and need more investigation (7–9).

A recent meta-analysis showed no significant difference in fatality rate and mechanical ventilation requirement between favipiravir treatment and the standard of care in 1,636 moderate and severe COVID-19 patients (9). In comparison, a retrospective observational study of 480 moderate to critical patients associated the favipiravir use with accelerated discharge rate and less progression to mechanical ventilation (10). Another meta-analysis of 827 patients revealed a significant clinical improvement in the favipiravir group vs. the control group during 7 days after hospitalization; however, favipiravir intake did not improve viral clearance, supplemental oxygen need, or mortality rate (11).

Therefore, we aimed to evaluate the safety and efficacy of favipiravir in hospitalized patients with moderate and severe COVID-19 using an observational cohort study design, where favipiravir was the primary available antiviral treatment.

## Methods

### Study Design and Participants

A prospective, multicenter observational study was conducted that included moderate and severe hospitalized adult COVID-19 patients in four major regions Riyadh (Riyadh), Eastern (Dammam), Al-Qassem (Buraydah), and Macca (Jeddah) of Saudi Arabia. Ethical approval was granted by the Medical Research Committee, Ministry of Health, Saudi Arabia. This study was approved by the Central Institutional Review Board in The Saudi Ministry of Health (MoH) with the approval number IRB # 20-85-M. Written informed consent was obtained from all human participants.

All consecutive patients ≥18 years of age admitted to hospitals with COVID-19 during a period from June 2020 and January 2021 were investigated in our study. COVID-19 diagnosis was confirmed by a positive SARS-CoV-2 infection polymerase chain reaction (PCR) test. Moderate COVID-19 was defined by PCR-positive COVID-19 requiring hospitalization, while severe COVID-19 was defined by PCR-positive hospitalized COVID-19 patients requiring respiratory support (10). Patients received either favipiravir (1,800 or 1,600 mg twice daily loading dose, followed by 800 or 600 mg twice daily) or supportive-care treatment. During the study period, favipiravir was the main antiviral medication available, and remdesivir was given only to few patients.

### Data Collection

A standard data collection was designed for the purpose of this study. Information was collected prospectively from the patient's medical files, laboratory data, and pharmacy data. Data on patients' demographics, vital signs, laboratory results, pharmacological treatments, and hospital outcomes were collected.

### In-hospital Outcomes

The primary outcome was all-cause mortality, and the secondary outcomes was the length of hospital stay.

### Statistical Analysis

Chai-square for categorical variables and Student *t*-test or Mann-Whitney *U*-test for continuous variables (where appropriate) were used to compare baseline clinical characteristics and interventions of patients who received favipiravir to those who did not.

For the primary outcome of all-cause mortality, a Cox proportional hazard analysis was performed. Time zero was the hospital admission date in all survival analyses. Moreover, favipiravir treatment was considered as a time-varying covariate to avoid “immortal time bias” or “survivor selection bias”, which occurs because patients who live longer are more likely to receive treatment than those who die early (8, 12, 13). The immortal time bias also considers the duration between initial symptoms and favipiravir intake. The hazard ratio with 95% CIs was used to estimate the all-cause mortality associated with favipiravir treatment. Kaplan–Meier survival curve was constructed to show cumulative survival over the hospital stay.

We also compared the length of hospital stay between favipiravir vs. no favipiravir treatment groups. We have used a generalized linear model to evaluate the length of hospital stay between these two treatment groups on the given days. In both Cox regression and general linear model analysis, we adjusted for age and gender, and dexamethasone and neutrophil to lymphocyte ratio, which were found to be significant in univariate analysis and were previously associated with COVID-19 outcome.

All tests were 2 tailed, and a *P*-value of < 0.05 was considered statistically significant. Statistical analysis was performed using SPSS (version 27.0), R software (version 3.6.1) and PRISM (version 8).

## Results

**Figure 1:**
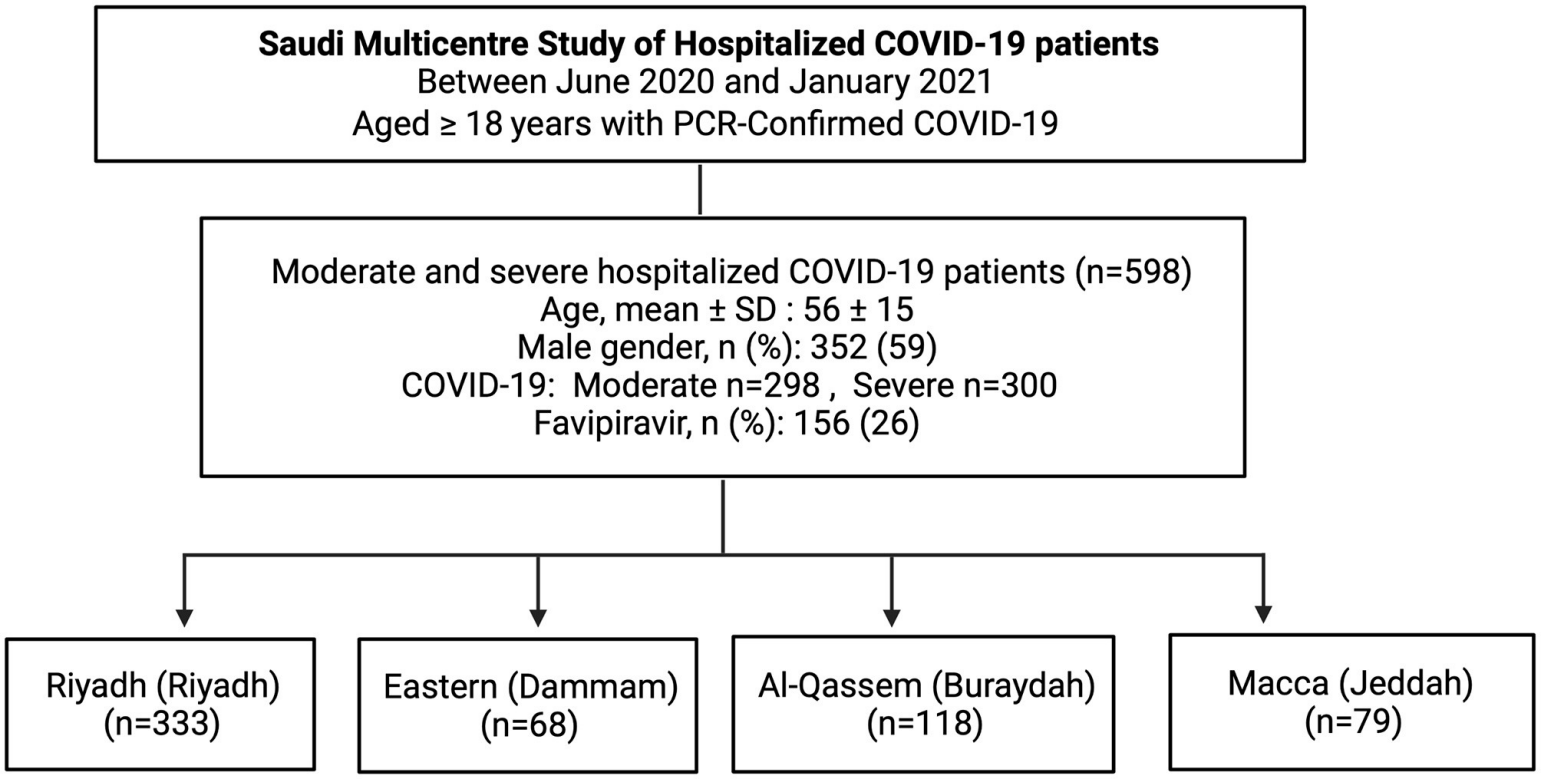
The flow chart of the study. SD, standard deviation.

Between June 2020 and January 2021, a total of 614 PCR confirmed COVID-19 patients were admitted to multiple hospitals located in four regions across Saudi Arabia. Of these, 16 patients did not have complete information, and they were excluded. The remaining 598 patients were included in our study, one-fourth of whom (*n* = 156, 26%) received favipiravir, while the remaining three-fourth (*n* = 442, 74%) received non-favipiravir standard care (Figure 1).

The study sample included 352 (59%) males and 246 (41%) females; the mean (SD) age of these patients was 56 ± 15, range 18–93. More than one-fourth (27%) of all patients were aged ≥65 years. Table 1 displays comparisons between clinical characteristics and hospital outcomes of favipiravir and no-favipiravir treatment groups.

**Table 1:** Baseline clinical characteristics of the study population.

**Variables**	**Total *n* = 598**	**No Favipiravir** ***n*** = **442**	**Favipiravir** ***n*** **156**	***P*** **-value**
**Age (years), mean ±SD**	56 ± 15	56 ± 16	56 ± 13	0.983
Age > 65		125 (28)	35 (22)	0.156
Male sex, *n* (%)	352 (59)	255 (58)	97 (62)	0.327
**BMI (kg/m**^**2**^**)**, ***n*** **(%)**				0.523
BMI 25–29.9	225 (38)	165 (37)	60 (39)	
BMI >30	259 (43)	188 (43)	71 (46)	
**Vital signs**				
Temperature (°C), median (IQR)	37 (1)	37 (1)	37 (1)	0.715
Oxygen Saturation (%), mean ± SD	93 ± 5	93 ± 5	93 ± 5	0.999
**Pre-existing** **conditions,** ***n*** **(%)**				
Hypertension, *n* (%)	244 (41)	181 (41)	63 (41)	0.947
Hyperlipidemia, *n* (%)	47 (8)	38 (9)	9 (6)	0.259
Diabetes, *n* (%)	272 (46)	203 (46)	69 (44)	0.714
Chronic liver diseases, *n* (%)	3 (0.5)	3 (1)	0 (0)	0.302
Chronic kidney diseases, *n* (%)	38 (6)	27 (6)	11 (7)	0.678
Other Cardiovascular, *n* (%)	70 (12)	57 (13)	13 (8)	0.128
Malignancy, *n* (%)	29 (5)	20 (5)	9 (6)	0.534
**Baseline** **laboratory data**				
White-cell count (10^9^/L), median (IQR)	6 (4)	6 (4)	7 (5)	0.473
**Differential** **count (10**^**9**^**/L)**				
Neutrophils	5 (4)	5 (4)	7 (7)	**0.003**
Lymphocytes	1.1 (0.8)	1 (1)	1 (1)	0.314
Monocytes	0.4 (0.4)	0.4 (0.4)	0.4 (0.5)	0.115
Neutrophil to lymphocyte ratio	4 (4)	4 (4)	5 (8)	**0.006**
Platelet count (10^9^/L)	213 (107)	211 (112)	216 (88)	0.719
Alanine aminotransferase (U/L)	45 (41)	31 (28)	39 (31)	0.806
Aspartate aminotransferase (U/L)	46 (110)	42 (26)	38 (32)	**<0.001**
Total Bilirubin (umol/L)	9 (4)	8 (5)	8 (5)	0.409
BUN (mmol/L)	9 (5)	5 (3)	6 (4)	0.417
Serum creatinine (UMOL/L)	55 (51)	76 (28)	80 (33)	0.251
**Pharmacological** **treatment**				
Azithromycin, *n* (%)	372 (62)	278 (63)	94 (60)	0.559
Dexamethasone, *n* (%)	147 (25)	43 (10)	104 (67)	**<0.001**
Hydroxychloroquine, *n* (%)	7 (1.2)	5 (1)	2 (1)	0.880
Interferon Beta, *n* (%)	1 (0.2)	1 (0.2)	0 (0)	0.552
Remdesivir, *n* (%)	8 (1)	7 (2)	1 (1)	0.378
**Hospital outcomes**				
**COVID-19 Severity**				**<0.001**
Moderate	298 (49.8)	194 (44)	104 (67)	
Severe	300 (50.2)	248 (56)	52 (33)	
**Hospitalization duration (days), median (IQR)**	9 (13.5)	10 (10)	14 (14)	**0.002**

*P-value of < 0.05 was considered as statistically significant*.

Age, gender, body mass index (BMI), admission temperature and oxygen saturation, and pre-existing conditions, were similar in favipiravir and no favipiravir users, as displayed in Table 1. Admission laboratory screening showed that aspartate aminotransferase was lower in the favipiravir group compared with those without favipiravir; 38 (32) vs. 42 (26) U/L (*P* < 0.001), while neutrophil to lymphocyte ratio was higher in favipiravir group; 5 (8) vs. 4 (4) (*P* = 0.006).

### Pharmacological Treatment

During the study period, the favipiravir was the main antiviral used for COVID-19 treatment; the other antiviral medication, remdesivir, was given only to one percent (*n* = 8) of patients. Patients with favipiravir were prescribed more dexamethasone (67 vs. 10%, *P* < 0.001), but Azithromycin was used similarly between the favipiravir users and non-users (Table 1).

### Adverse Effects Comparison in Favipiravir Users and Non-users

We compared the gastrointestinal, renal, and liver adverse effects in favipiravir users and non-users during the first 2 weeks of the medication intake. Compared to non-users, favipiravir intake was associated with more diarrhea (12 vs. 5%, *P* = 0.002), while there was no difference in renal and liver profiles between favipiravir and no favipiravir groups (Table 2).

**Table 2:** Adverse effects and liver and renal functional test in favipiravir treatment and control group.

**Variables**	**No Favipiravir** ***n*** **= 442**	**Favipiravir** ***n*** **= 156**	***P*** **-value**
Nausea, *n* (%)	29 (7)	9 (6)	0.727
Vomiting, *n* (%)	22 (5)	13 (8)	0.125
Diarrhea, *n* (%)	22 (5)	19 (12)	**0.002**
**Alanine aminotransferase (U/L), median (IQR)**			
Weak 1 (*n* = 391)	34 (37)	40 (49)	0.051
Weak 2 (*n* = 180)	49 (51)	47 (44)	0.887
**Aspartate aminotransferase (U/L), median (IQR)**			
Weak 1 (*n* = 314)	43 (29)	38 (37)	0.284
Weak 2 (*n* = 141)	33 (23)	29 (15)	0.556
**Total Bilirubin (μmol/L), median (IQR)**			
Weak 1 (*n* = 391)	8 (4)	8 (5)	0.439
Weak 2 (*n* = 184)	8 (5)	8 (6)	0.839
**Serum creatinine (UMOL/L), median (IQR)**			
Weak 1 (*n* = 441)	73 (26)	72 (31)	0.703
Weak 2 (*n* = 218)	70 (24)	70 (51)	0.695

*P-value of < 0.05 was considered as statistically significant*.

### In-hospital Outcomes

**Figure 2:**
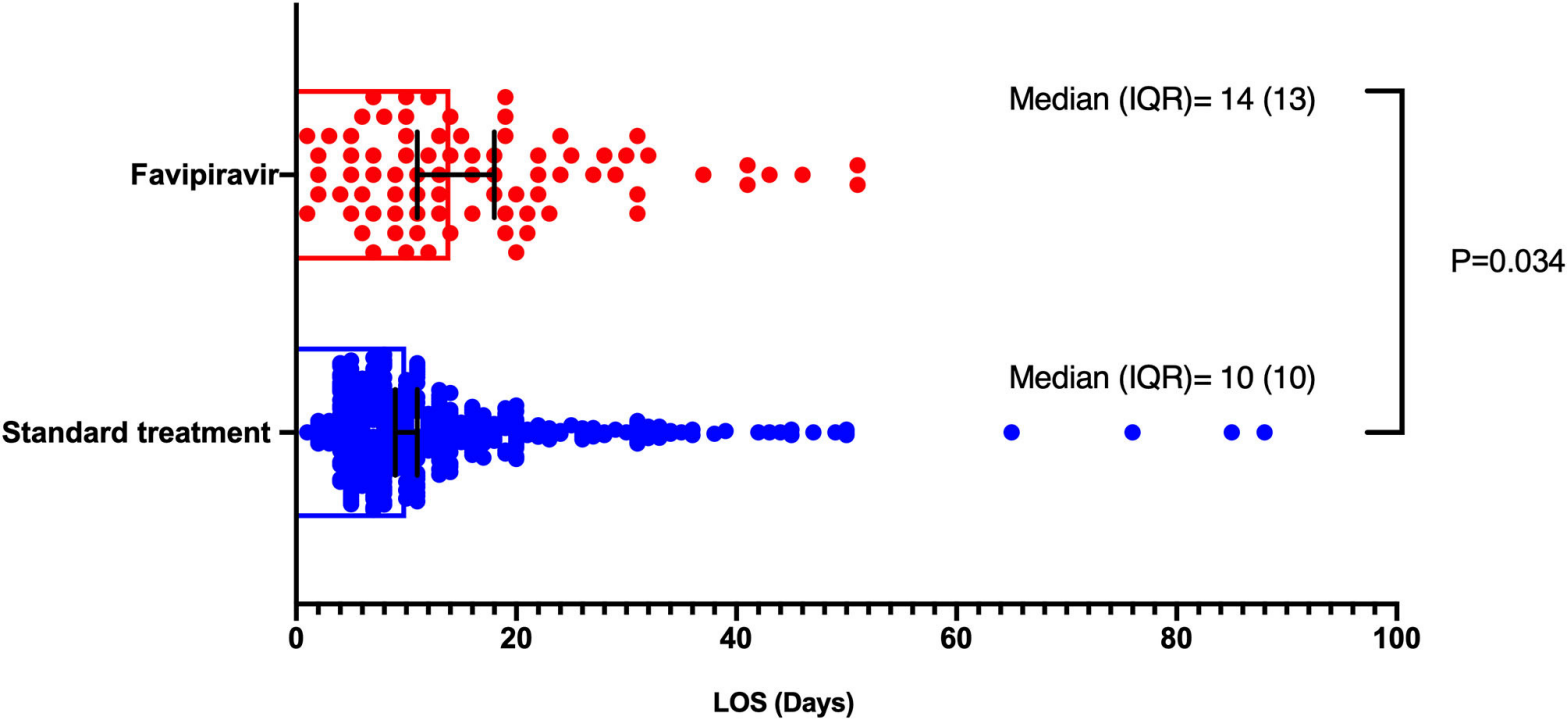
Length of hospital stay comparison between favipiravir and no favipiravir groups. Generalized linear models were used, which was adjusted for age, gender, dexamethasone, and neutrophil to lymphocyte ratio.

Short-term outcomes of length of hospital stay and all-cause mortality were compared between favipiravir users and non-users. In univariate analysis, Favipiravir use was associated with longer hospital stay; the median length of hospital stay was 14 days (IQR 13) group with favipiravir vs. 10 (IQR 10) in those with no favipiravir. In a generalized linear model adjusted for age, gender, dexamethasone, and neutrophil to lymphocyte ratio, favipiravir intake was associated with longer hospital stay (*P* = 0.034), as displayed in Figure 2.

**Figure 3:**
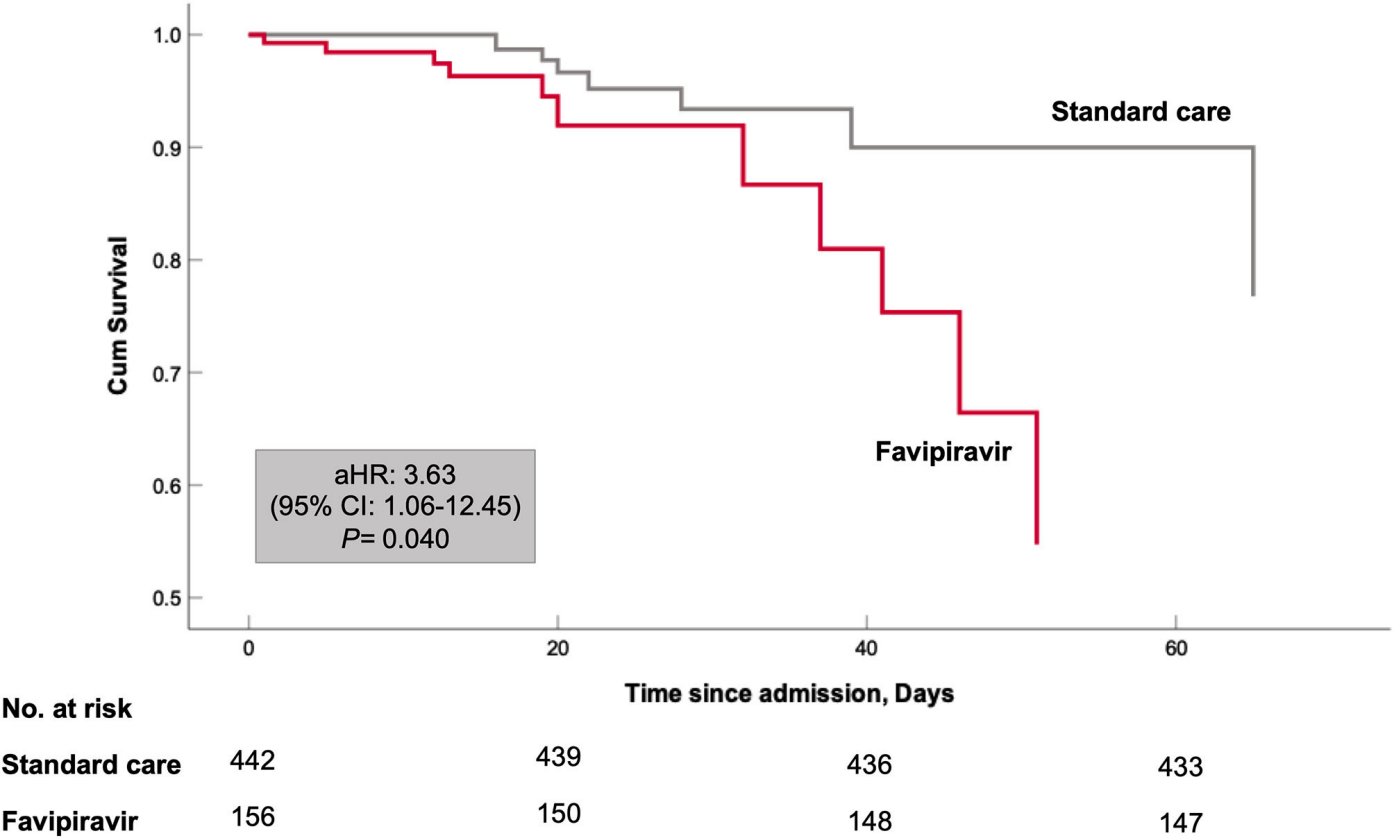
Kaplan–Meier curves for in-hospital survival. Cox proportional model has been adjusted for both patient baseline variables at admission (patients' age, male sex, body mass index) and patient COVID-19 related variables during hospital stay (Neutrophil to lymphocyte ratio and systemic use of dexamethasone).

To assess the risk of all-cause mortality, Cox regression analysis was performed. Age and gender-adjusted model showed higher all-cause mortality in favipiravir users [adjusted hazard ratio (aHR) 3.47; 95% confidence interval (CI) 1.24–9.71; *P* = 0.018]. Further adjustment with neutrophil to lymphocyte ratio and dexamethasone variables did not change this association [aHR 3.63; 95% CI 1.06–12.45; *P* = 0.034]. Kaplan–Meier curve for this model is shown in Figure 3. Immortal time bias was accounted for in the Cox regression analysis by considering medications as time-dependent variables (8, 12). The results of the survival analysis are displayed in Table 3.

**Table 3:** Survival analysis for all-cause mortality.

	**Crude HR (95% CI)**	Adjusted HR (95% CI) (age + gender)*	Adjusted HR (95% CI) (age + gender, + NLR + dexamethasone)*
Favipiravir	5.15 (2.24–11.83)	3.47 (1.24–9.71)	3.63 (1.06–12.45)

* *Immortal time bias was accounted for in the Cox proportional model by considering medications as time-dependent variables*.

## Discussion

In this prospective observational cohort study of patients hospitalized with moderate to severe COVID-19, favipiravir treatment was associated with more extended hospital stay (14 vs. 10 median, *P* = 0.034) and higher mortality rate (aHR 3.63; 95% CI 1.06–12.45) as compared to no favipiravir regimen. Despite lack of effectiveness, favipiravir intake was only associated with higher diarrhea adverse effects (12 vs. 5%, *P* = 0.002), but it did not affect the renal and liver profile of patients.

In general, favipiravir has shown a consistent safety profile with no significant adverse events compared to the no favipiravir group in the recommended dose range (6, 11, 14). While in terms of efficacy in moderate and severe COVID-19 conflicting results were reported (9–11). The initial endorsement of favipiravir was based on its effect to reduce viral clearance (14, 15), however later in the pandemic, viral clearance was shown not to be the proper measure of medication effectiveness, with many patients continuing to have positive RT-PCR results even after complete recovery (9, 16).

In our study, we evaluated two outcomes of length of hospital stay and in-hospital mortality. Favipiravir did not reduce the length of hospital stay in our moderate and severe cohort. This result was partly following the result of a retrospective report by Alamer et al., which showed that while favipiravir effect on reducing the time to discharge was significant in severe to critical patients, its effect was not significant in a moderate group (10). The difference in severe and critical results could be due to retrospective design and different analysis approaches used by Alamer et al. to account for missing variables (10). Nevertheless, both studies showed favipiravir did not reduce the hospital length in moderate COVID-19. The second outcome of in-hospital mortality reduction by favipiravir was consistently not significant in our study, and both Alamer et al. and Malhani et al. reports (8, 10).

Lack of effectiveness in moderate and severe cases could also be explained by the timing of treatment administration, the dosing regimen prescribed, or both. Driouich et al. investigated the time and dosing of favipiravir using *in vivo* Syrian hamster model (17). In their study, authors showed that when treatment was initiated before or simultaneously to infection, favipiravir presented with a strong dose effect, leading to reduction of infectious titers in lungs and clinical alleviation of the disease. Additionally, favipiravir antiviral activity against SARS-COV-2 infection in the hamster model was established in the higher dose range, which was also followed by a sign of toxicity (17). Confirming the hamster model study, another population pharmacokinetic analysis of favipiravir revealed that compared to healthy volunteers, the concentration of this medicine was found to be much lower in critically ill patients with COVID-19 who required invasive mechanical ventilation (18). Further simulation analysis indicated that that the 1,600/600 mg BID regimen may be insufficient for the treatment of COVID-19 (18).

Altogether, the lack of effect observed in our study and the majority of previous moderate and severe COVID-19 investigations suggest that favipiravir could be more effective as prophylactic or early in the course of infection. Moreover, the dosing regimen may need optimization to achieve maximum effectiveness in SARS-COV-2 eradication while inducing tolerable toxicity. The dose might also need to be personalized based on patient factors such as total body surface area. Further pharmacokinetic and clinical studies are needed to optimize favipiravir repurposing for SARS-COV-2 viral infection.

## Strength and Limitations

Our study has multiple strengths: First, this study was a multicenter observational study in Saudi Arabia. Second, although this was observational, our study was less susceptible to treatment section bias, and treatment allocation was based on favipiravir availability and national management guidelines. Third, our analysis accounted for immortal time bias. Not considering the immortal time in observational studies could lead to inflated treatments' effect estimates (8, 12). Our study has some limitations. Due to the observational nature, we could not completely rule out the effect of potential confounding or treatment selection bias. In addition, several events limited our ability to adjust for more potential confounders in the Cox model. However, we adjusted for dexamethasone use and neutrophil to lymphocyte ratio factors that were significant in univariate analysis, and they were strong potential cofounders based on previous COVID-19 literature (19–21).

## Conclusions

In this multicenter observational prospective study of moderate-severe COVID-19 hospitalized patients, we observed that favipiravir was not effective in reducing the length of hospital stay and in-hospital mortality.

## Data Availability Statement

The original contributions presented in the study are included in the article/supplementary material, further inquiries can be directed to the corresponding author/s.

## Ethics Statement

The studies involving human participants were reviewed and approved by Central Institutional Review Board in The Saudi Ministry of Health (MoH) approval number (IRB # 20-85-M). The patients/participants provided their written informed consent to participate in this study.

## Author Contributions

HA-S, RH, SA-M, NA-N, MA, FA, and NS conceived and designed the study. RB, BA, and AJ collected the data. NS, FS, and RH analyzed the data. All authors contributed to writing and revision of the manuscript.

## Funding

The authors extend their appreciation to the Deputyship for Research and Innovation, Ministry of Education in Saudi Arabia for funding this research work through the project number (DRI-KSU-928).

## Conflict of Interest

HA-S was employed by Hevolution Foundation. The remaining authors declare that the research was conducted in the absence of any commercial or financial relationships that could be construed as a potential conflict of interest.

## Publisher's Note

All claims expressed in this article are solely those of the authors and do not necessarily represent those of their affiliated organizations, or those of the publisher, the editors and the reviewers. Any product that may be evaluated in this article, or claim that may be made by its manufacturer, is not guaranteed or endorsed by the publisher.
